# Construction and Operation of a Ventilated Hood System for Measuring Greenhouse Gas and Volatile Organic Compound Emissions from Cattle

**DOI:** 10.3390/ani1040433

**Published:** 2011-12-08

**Authors:** Sara E. Place, Yuee Pan, Yongjing Zhao, Frank M. Mitloehner

**Affiliations:** Department of Animal Science, University of California, Davis, One Shields Ave., Davis, CA 95616, USA; E-Mails: seplace@ucdavis.edu (S.E.P.); yepan@ucdavis.edu (Y.P.); yjzhao@ucdavis.edu (Y.Z.)

**Keywords:** eructation, cattle, emissions, greenhouse gas, measurement

## Abstract

**Simple Summary:**

We describe the construction and operation of a unique system for measuring gaseous emissions that arise from the rumen and metabolism of cattle. This system allows for the collection of high quality data that can be used to improve emission inventories at the regional and national level. Additionally, the system can be used to test various emission mitigation techniques.

**Abstract:**

Recent interest in greenhouse gas emissions from ruminants, such as cattle, has spawned a need for affordable, precise, and accurate methods for the measurement of gaseous emissions arising from enteric fermentation. A new head hood system for cattle designed to capture and quantify emissions was recently developed at the University of California, Davis. The system consists of two head hoods, two vacuum pumps, and an instrumentation cabinet housing the required data collection equipment. This system has the capability of measuring carbon dioxide, methane, ethanol, methanol, water vapor, nitrous oxide, acetic acid emissions and oxygen consumption in real-time. A unique aspect of the hoods is the front, back, and sides are made of clear polycarbonate sheeting allowing the cattle a full range of vision during gas sampling. Recovery rates for these slightly negative pressure chambers were measured ranging from 97.6 to 99.3 percent. This system can capture high quality data for use in improving emission inventories and evaluating gaseous emission mitigation strategies.

## 1. Introduction

In recent years, there has been a heightened awareness of global climate change, and a corresponding increased scrutiny of anthropogenic sources of greenhouse gases (GHG). Animal agriculture, and specifically ruminant livestock production (e.g., cattle, sheep, goats, *etc.*), has been identified as a major contributor of GHG worldwide [1]. A significant portion of GHG emissions from US animal agriculture result from microbial processes in ruminant digestive tracts (an estimated 20 percent of methane emissions and 2.1 percent of total US GHG emissions in 2009 were attributed to enteric fermentation by [2]). Model-derived estimates of GHG emissions from the fore-stomachs of ruminants often lack the accuracy required to produce valid inventories [3] and data sets derived from live animal experiments are needed for their improvement. Additionally, predicting the effectiveness of emission mitigation techniques requires collection of accurate and precise data from live-animal experiments. Techniques and equipment used to collect such data vary, but one particularly effective apparatus for measurement of gaseous emissions arising from the rumen of cattle and respiration is the use of an open-circuit ventilated hood system. This type of system has a cost advantage over whole-animal respiration chambers, and an accuracy advantage over the sulfur hexafluoride measurement technique when considering methane emissions [4].

While these ventilated hood-type systems have been employed for decades in animal metabolism studies, the increased awareness of the environmental impact of ruminants has driven a renewed interest in constructing and refining such systems to calculate emission rates and test mitigation strategies of air pollutants [5]. Virtually all previous ventilated hood-type systems have measured the gases carbon dioxide, oxygen, and methane exclusively, but it is possible that numerous other gas species originate from the rumen, which are currently or likely to be regulated by government agencies in the future (e.g., nitrous oxide, methanol, ethanol, *etc.*). Volatile organic compounds (VOC) are of particular interest in the state of California, as these compounds can react with oxides of nitrogen in the presence of sunlight to form ozone. Ozone levels are often above the 8-hour National Ambient Air Quality Standard of 75 ppb in certain California air sheds, in particular the San Joaquin Valley that is home to over 1.6 million dairy cattle [6]. While it is now known that many of the VOC from animal agriculture originate from fermented feeds (e.g., silage) [7], no previous systems measuring emissions strictly arising from the rumen have had the capability to measure these compounds in real-time and calculate emission rates. A new ventilated hood-type emissions measurement system that has the capability to measure these and other gases eructated from cattle was recently developed at the University of California, Davis. The following article will describe the design, construction, and operation of this system in detail to provide a template for future researchers to use and improve upon.

## 2. System Design and Components

### 2.1. Overview

**Figure 1:**
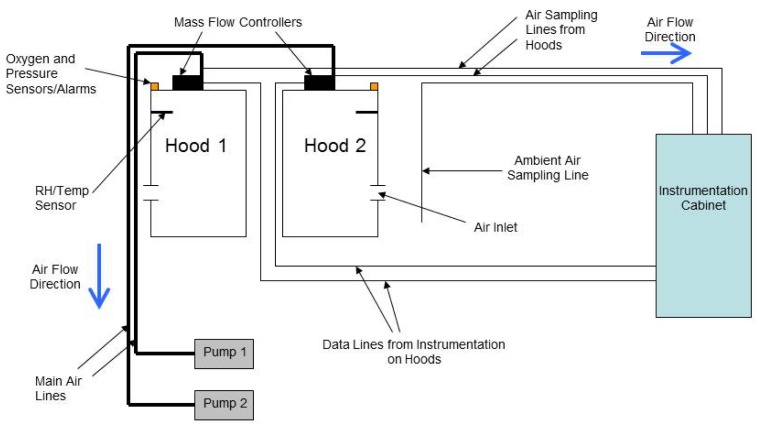
A schematic that depicts an overview of the open-circuit ventilated hood system described.

**Figure 2:**
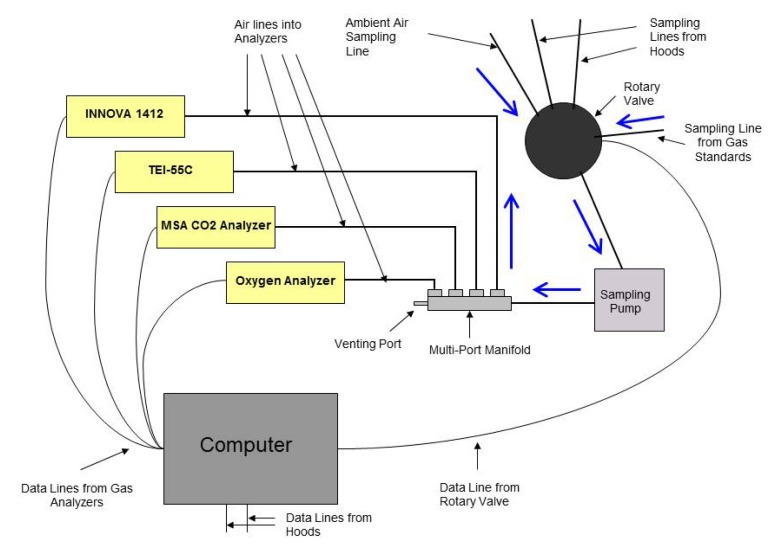
A schematic overview of the set-up inside the instrumentation cabinet. Blue arrows indicate the air flow direction.

The system consists of two head hoods, two large vacuum pumps used to evacuate air from the hoods, and an instrumentation cabinet that houses the gas analyzers, computer, sampling pump, *etc.* Diagrams of the overall set-up and the instrumentation inside the cabinet can be seen in Figure 1 and Figure 2. The following sections will describe in detail the dimensions and materials used to construct the hoods and the measurement equipment that is required to calculate emission rates from cattle.

### 2.2. Head Hoods

The two head hoods designed for this system were constructed at University of California, Davis. The basic dimensions and design of the head hood was similar to those of Odongo *et al.* [5] and Kelly *et al.* [8]; however, modifications were made and some of the materials used were unique. The hood was 151 cm × 104 cm × 76.2 cm (H × W × D) and on the back, front, and two sides of the hood, 0.635 cm clear polycarbonate sheeting was used to provide a full range of vision for the cow inside. The top and bottom of the hood was made from 1.90 mm thick stainless steel sheeting. The frame of the hood was made of 3.81 cm × 3.81 cm × 4.76 mm stainless steel angle iron. The polycarbonate sheeting was laid on the outside of the angle iron frame. On one side of the head hood, a 10.2 cm diameter inlet port was cut in the polycarbonate sheeting to allow outside air to enter the hood during gas sampling. On the same side of the hood, a water bowl was attached 50.8 cm above the base to two support pieces of stainless steel flat iron. On the front of the hood was a door that could be opened when the cow was placed in the hood and to offer the animal feed. The door was sealed during gas sampling by three locking catches on top, four locking catches on the side, and two screws on the bottom of the door. Foam tape was placed on the edge of the door frame to ensure an adequate seal.

On the back of the hood, a 73.7 cm wide and 114 cm tall oval neck opening was cut into the sheeting to allow the cow's head to enter the hood. A neck sleeve was attached to this opening and placed over the cow's head and neck to minimize the air leaking into the hood. The neck sleeve was made from a black military strength tarp material tapered to a 122 cm circumference at the end that attached to the cow's neck. The neck sleeve had a plastic buckle and strap that could be adjusted at the end attaching to the cow's neck and Velcro on the entire sleeve up to 25.4 cm from the hood. The end of the sleeve that was attached to the hood had a 3.18 mm diameter steel cable inserted and a circumference of 302 cm. This end of the sleeve was then attached to the head hood by fitting it over rolled steel that surrounded the neck opening of the hood, and then placing two pieces of molded steel rod over the material. The two pieces of 0.635 cm diameter stainless steel rod could then be attached together and tightened via a 0.953 cm × 8.89 cm bolt on either side of the oval-shaped neck opening.

**Figure 3:**
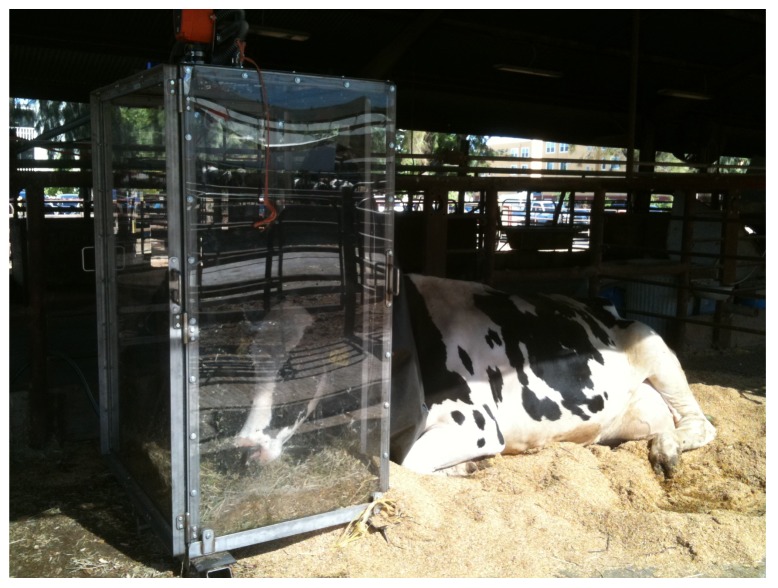
An example of a cow lying down while her eructated and respired emissions are being measured in the head hood.

**Figure 4:**
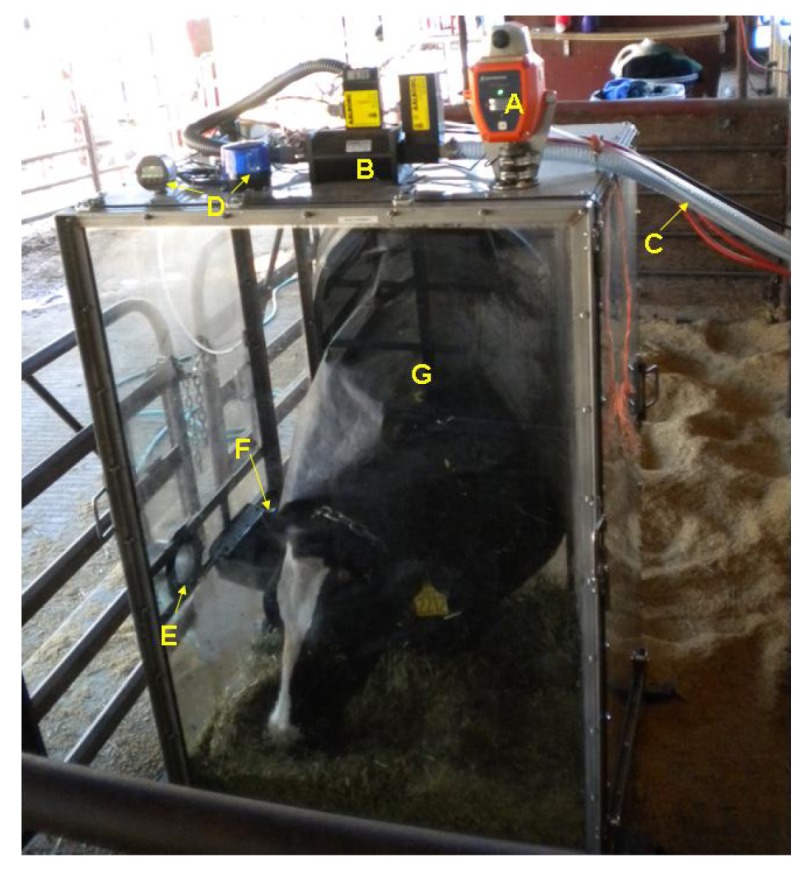
A dairy cow standing and eating during a gas sampling period. **(A)** is the oxygen sensor/alarm; **(B)** is the mass flow controller; **(C)** is the main air line that was connected to the rotary vane vacuum pump; **(D)** is the differential pressure gage/alarm system; **(E)** is the air inlet opening; **(F)** is the water bowl for the cow; **(G)** is the neck sleeve.

On the floor of the hood, a chain was attached to the two sides of the base and a tie strap with a clip on the end was attached in the middle of the chain. When a cow entered the hood, her chain necklace was attached to this tie strap via the clip, which allowed a full range of movement so the cow could easily eat, drink, lie down, and get up if she desired. Figure 3 and Figure 4 are photographs of the ventilated head hoods during data collection from lactating Holstein dairy cattle.

On the top of the hood was a 2.54 cm diameter opening that was used as the air outlet. On the side of the extrusion of this outlet was a Swagelok fitting for the connection of the outlet sampling line. The head hood had an oxygen sensor/alarm and pressure sensor/alarm located on the top of the hood (Sperian Biosystems NXP fixed mount gas detector from Cole-Parmer, Vernon Hills, IL, USA and DPG-200 from Dwyer Instruments, Inc., Michigan City, IN, USA, respectively). Located on the upper part of the same side of the hood that housed the inlet opening and water bowl, the hood had a relative humidity/temperature sensor/transmitter with a duct mount (Setra Systems, Boxborough, MA, USA) that was connected to a computer for real-time acquisition of the data.

### 2.3. Gas Sampling System

The air inside the head hood exited the outlet on the top of the hood and was drawn via a large rotary vane vacuum pump (Model 6066-V107A-T339 equipped with a 5 HP electric motor, GAST Manufacturing, Inc., Benton Harbor, MI, USA) that supplied approximately 650 L/min air flow rate. The air was pulled from the outlet on top of the hood through 2.54 cm I.D. × 3.72 cm O.D. Polywire Plus steel-braid reinforced tubing and first entered a mass flow controller (AALBORG, Orangeburg, NY, USA) that was secured on top of the head hood. The mass flow controller had an analog signal indicating the air flow rate, and real time output of the flow rate at standard temperature and pressure was digitized, recorded and graphed on the computer inside the instrumentation cabinet.

Air samples were taken from the outlet of each hood and the ambient air from a location adjacent to the hoods. Air samples were drawn through crack-resistant Teflon PFA tubing measuring 3.18 mm I.D. × 6.35 mm O.D. × 1.59 mm wall thickness by a 1/20 HP vacuum/pressure PTFE-coated diaphragm pump (Cole Parmer, Vernon Hills, IL, USA) located inside the instrumentation cabinet. The three sampling lines entered an 8-position micro electric rotary actuator valve (Valco Instruments Co., Inc., Houston, TX, USA) that was controlled by the computer to switch from drawing air samples from each of three locations to the gas analyzers every 20 minutes. The five unused ports on the rotary valve were plugged. An orifice was constructed in the outlet of the sampling pump to restrict the sampling air flow rate to approximately 7 L/min.

**Figure 5:**
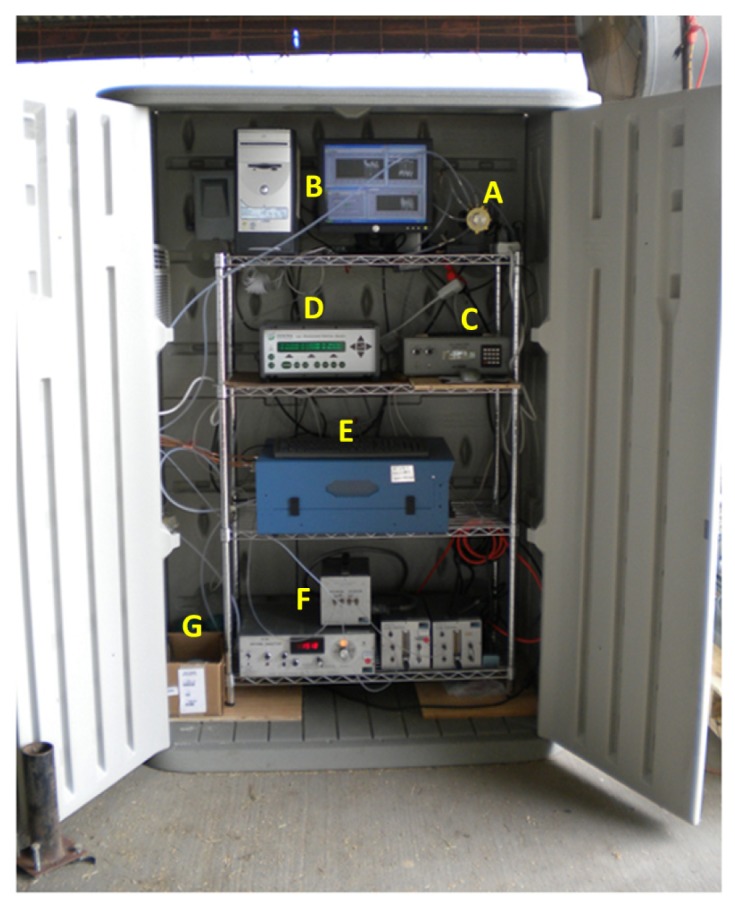
The instrumentation cabinet open while cattle were in both of the hoods. **(A)** is the rotary valve. **(B)** is the computer and monitor displaying the real-time data output of gas concentrations, RH/temp, and air flow rate. **(C)** is the carbon dioxide analyzer. **(D)** is the photoacoustic field gas monitor (INNOVA 1412). **(E)** is the direct methane/non-methane hydrocarbon analyzer (TEI-55C). **(F)** is the oxygen analyzer (sensor, readout/control unit, and 2 flow controllers (with pump, needle valve, and flow meter)). **(G)** is the sampling pump.

**Figure 6:**
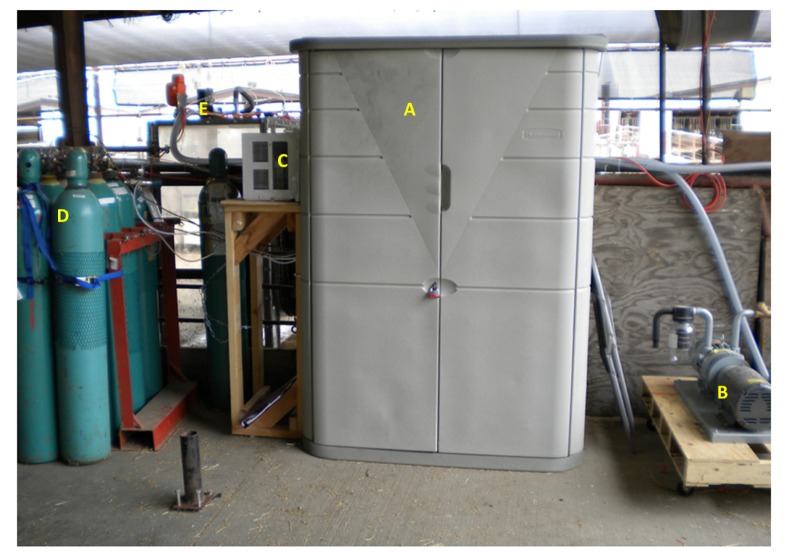
A photo of the instrumentation cabinet closed. **(A)** is the closed instrumentation cabinet housing the gas analyzers. **(B)** is one of 2 rotary vane vacuum pumps powered by 5 HP electric motors. **(C)** is an A/C unit used to cool the inside of the instrumentation cabinet. **(D)** are cylinders used for calibration of some of the analyzers, and as carrier and fuel gases for the TEI-55C analyzer. **(E)** is one of the head hoods in operation.

Once the air sample from one of the three locations was drawn through the sampling pump, it entered a multi-port manifold which was connected to tubing lines that went into each of the four gas analyzers. One port was exposed to ambient to release the excess sampling air so that the sampling line pressure was the same as the ambient pressure. An INNOVA 1412 photoacoustic gas analyzer (LumaSense Technologies, Ballerup, Denmark) was used to measure the concentrations of carbon dioxide, nitrous oxide, methanol, ethanol, acetic acid, and water vapor. All six gases were measured approximately every 60 seconds. A second set of back-up carbon dioxide concentration data was obtained using an MSA Model 3600 infrared carbon dioxide monitor (Mine Safety Appliances Company, Pittsburg, PA, USA). The oxygen concentration data was obtained using an S-3A oxygen analyzer (AEI Technologies, Inc., Naperville, IL, USA) with the oxygen concentration being determined every 0.1 seconds. Methane concentrations were determined with a TEI-55C direct methane, non-methane hydrocarbon analyzer (Thermo Environmental Instruments, Inc., Franklin, MA, USA) measured approximately every 70 seconds. The detection limits of these gas analyzers (according to the manufacturers' specifications) are summarized in Table 1. Both the INNOVA 1412 and the TEI-55C analyzers send digital output signals through an RS232 serial communication port, which were directly acquired by the computer. The MSA Model 3600 infrared carbon dioxide monitor and the S-3A infrared oxygen analyzer send analog output signals, which were digitized by an A/D converter before being recorded by the computer. The oxygen signals were also used to trigger “low-oxygen” alarm by sending registered users an e-mail with an alert message if oxygen concentrations dropped below 18 percent. A snap shot of the computer screen showing data graphs and measurement status was sent to the registered users hourly, so that the analytical equipment could be remotely monitored. All of the gas analyzers, the sampling pump, computer, and rotary valve were housed in the instrumentation cabinet that was kept at or below 20 °C via a small window A/C unit located on the side of the cabinet. Figure 5 and Figure 6 are photographs of the gas sampling/analyzing set-up.

## 3. System Operation

### 3.1. Validation and Recovery Rate

Prior to using the system for any live animal experiments, the ventilated hood system was tested for its accuracy and precision, and the recovery rate for each of the hoods was calculated. Both a polar (ethanol) and a non-polar gas (carbon dioxide) were used during the recovery rate experiment.

**Figure 7:**
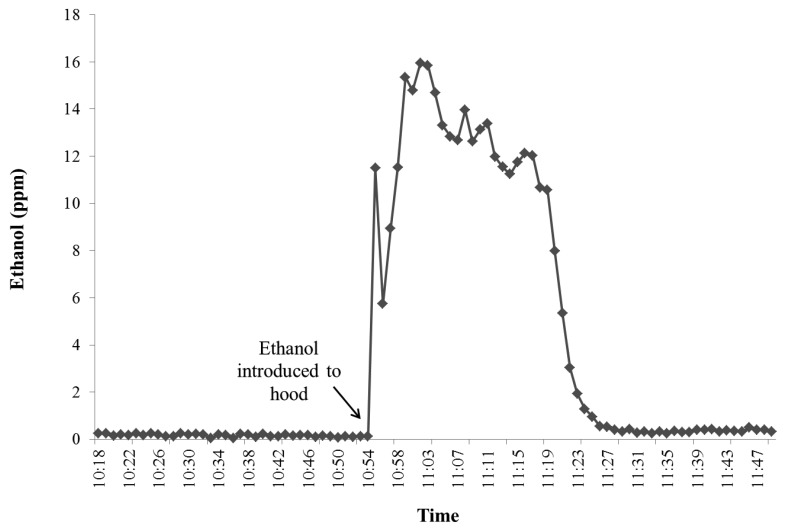
Ethanol concentration (ppm) over time during recovery rate test for head hood #2.

Table 2 contains a summary of the total injected and recovered gases for each of the two hoods and their corresponding recovery rates. For the validation experiment with ethanol, 0.5 mL of 99.5 percent aqueous ethanol was injected into the water bowl inside the hood while the vacuum pump was running for each hood. Virtually all of the ethanol evaporated within one hour as can be seen in Figure 7.

**Figure 8:**
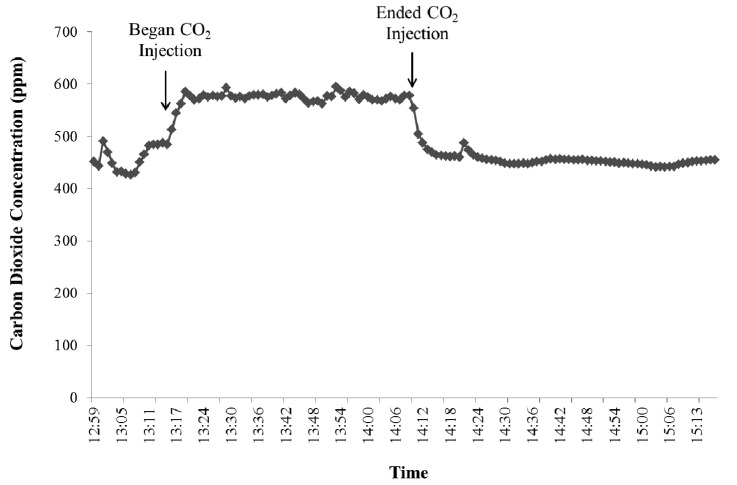
Carbon dioxide concentration (ppm) over time during recovery rate test for head hood #1.

For the validation experiment with carbon dioxide, a 1 percent carbon dioxide standard gas was introduced into each of the two hoods through the inlet opening with a constant flow rate. Background concentrations of carbon dioxide were accounted for by gas sampling before and after the carbon dioxide injection. Figure 8 depicts the carbon dioxide concentrations measured in hood #1.

The calculated recovery rates for the hoods ranged from 97.6 to 99.3 percent, which indicates that there was minimal loss of gas emissions inside the hood. The rates are very similar to previously reported recovery rates for similar systems, such as Suzuki *et al.* [9] who constructed four head hoods for cattle with recovery rates ranging 96.5 to 101.8 percent.

### 3.2. Example of Data During Live Animal Experiment

To calculate the emissions from the cattle in the head hoods, the gas concentrations at the outlet of each hood, the gas concentrations of background or ambient air, and the air flow rate through the hoods, must be known. The total volume of the hoods and the venting flow rate determine the turnover time, which is an indication how long the inside air can be completed replaced. Each head hood had an approximate volume of 1,200 L, which means that during gas sampling when the ventilation rate was close to 650 L/min, the hoods reached a steady-state within two minutes (the hood residence time). The turnover time of the hoods was approximately 5.5 minutes (three times the hood residence time).

**Figure 9:**
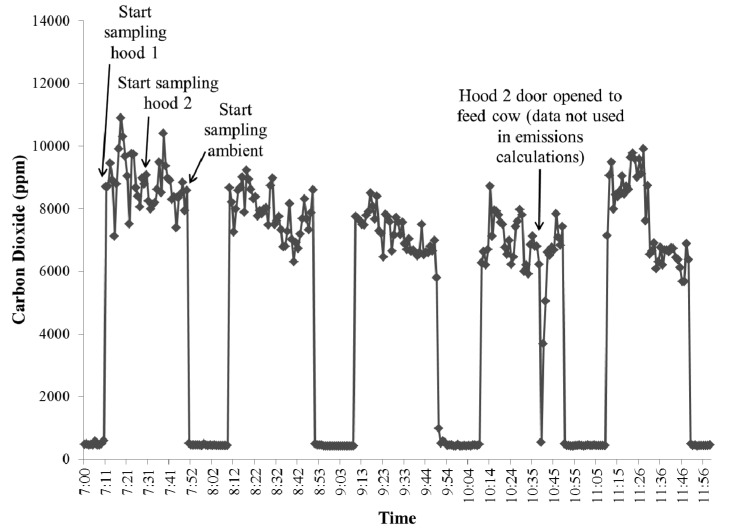
Example of raw carbon dioxide concentrations (ppm) over time from the INNOVA 1412.

**Figure 10:**
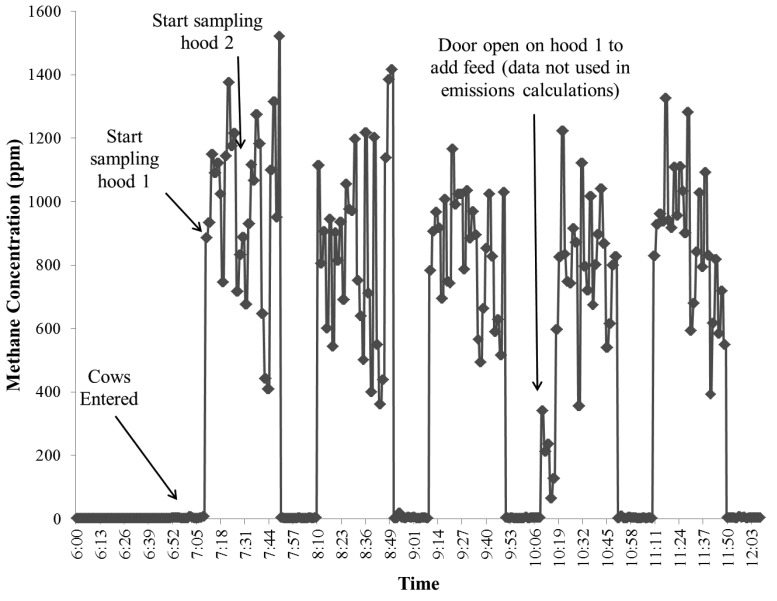
Example of raw methane concentrations (ppm) over time from the TEI-55C.

Figure 9 is an example of the raw carbon dioxide output data from the INNOVA 1412 and Figure 10 shows an example of the raw methane concentration data from the TEI-55C. The data is from an experiment during which the cattle were first placed in the hoods at approximately 07:00. It is important to mention that cattle being sampled with this type of system must be properly trained and adapted to spending time inside the hood to ensure the quality of the data (*i.e.*, cattle are not hyperventilating). The concentration peaks in Figure 9 and Figure 10 are when the sampling location was the outlet on either of the hoods, and the times in between those peaks are when the sampling location returned to the ambient air. The sampling time at each location was 20 minutes. The data from the first five minutes of sampling after the valve switched locations was not used to account for the flushing out of the sampling lines of the previous location's air. Ambient and outlet air concentrations were averaged over one hour intervals to calculate the volume emission rate (ER) at standard temperature and pressure:

ER=Net concentration(ppm)×Simpling flow rate(L/minute)

The net concentration of each gas was the difference between the outlet gas concentration and the ambient gas concentration. Emission rates in mass of a specific gas compound cow^-1^ hour^-1^ could then be calculated after accounting for temperature, pressure, and the mass of the compound of interest.

**Figure 11:**
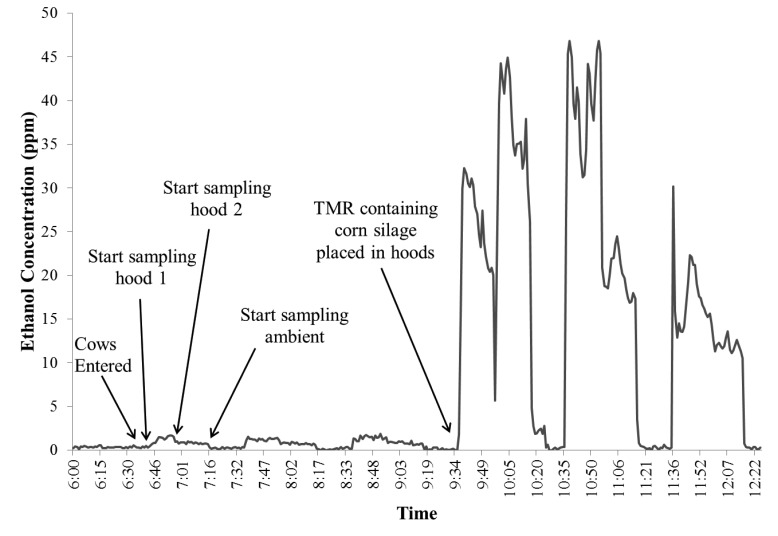
Example of raw ethanol concentrations (ppm) over time from the INNOVA 1412.

Figure 11 is an example of the raw ethanol output data from the INNOVA 1412. Dairy cows in the trial in which this data is from were feed a total mixed ration containing corn silage. As previously mentioned, fermented feeds such as silage are known sources of VOCs like ethanol [7], so the cows were fed dry alfalfa hay (90 percent dry matter) for approximately the first 3 hours of the sampling period before the silage-containing TMR was offered. As can be seen in Figure 11, there were clear increases in ethanol concentration when the sampling location was either hood outlet prior to adding the TMR, but there was a dramatic increase in ethanol concentrations once the silage was added that decreased over time.

Table 3 compares some of the recent published methane emission data from lactating Holstein dairy cows to the emissions found in an unpublished trial with the current system. The upper range for emission rates found with the current system were higher than others, but the upper range of dry matter intakes were also higher than others, which equated into similar methane-to-dry matter intake ratios. It is important to note that there were differences in factors known to affect methane emissions across the studies in Table 3, such as dietary fat content, forage percentage, stage-of-lactation, and the presence of feed additives, so the variability in emission rates is expected.

As mentioned previously, it is important to properly adapt the cattle to the measurement system before an experimental trial begins, which requires placing the animals inside the hood for increasing periods of time to increase their comfort with the system. However, a challenge with this and other emission measurement systems, such as whole animal chambers and sulfur hexafluoride systems that require animals to wear harnesses, is the researchers can never completely ensure that the animals being measured are not stressed in any way, or that the emission rates calculated with these systems accurately represent emissions from animals in their “normal” states on pasture, in pens in free-stall housing, in a tie-stall, *etc.* Of course, physiological measures can be taken, such as respiration rate and blood samples to test for stress hormones concentrations, to determine if animals are stressed; however, the issue remains that to obtain precisely measured emission data from individual or small groups of animals, some type of restraint and/or obstruction to the animal's normal routine is required. We considered this in our construction of this system, which is why the hood was constructed of clear polycarbonate sheeting: the animals may be restrained, but they can clearly see their surroundings, which was not the case with the ventilated hoods systems of Odongo *et al.* [5] and Kelly *et al.* [8]. Additionally, as can be seen in Table 3, the methane emission rates obtained with this system are in agreement with others who used whole animal chambers, ventilated hoods, and the sulfur hexafluoride technique, indicating that even if our system is altering emissions from cattle in their “normal” state, it is doing so in a way that is equivalent with the numerous emissions mitigation systems in existence.

## 4. Conclusions

The system described above offers a lower cost alternative to whole-animal respiration chambers and a measurement technique that allows researchers insight into the emissions arising directly from the rumen of cattle. The detailed datasets that can be obtained from this and similar systems will be critical for future inventory estimates of GHG and other pollutants at the national and regional level. With such a system, more precise measurements of the effect of potential mitigation strategies are possible. If combined with investigations of rumen microflora and chemistry, such a system can increase both knowledge of how the rumen ecosystem functions and how the microbial population of the rumen drives the emissions released from the rumen during eructation.

## Figures and Tables

**Table 1:** Summary of the gas analyzers used for the ventilated hood system.

**Analyzer model**	**Manufacturer**	**Detection method**	**Target gas**	**Detection Limit**
INNOVA 1412	LumaSense Technologies, Ballerup, Denmark	Infrared photoacoustic	CO_2_ Methanol Ethanol N_2_O Acetic Acid	1.5 ppm 0.14 ppm 0.08 ppm 0.03 ppm 0.1 ppm
TEI 55C	Thermo Electron Corp., Franklin, MA, USA	Back-flush Gas chromatography	CH_4_	20 ppb
MSA 3600	Mine safety Appliances, Co., Pittsburgh, PA, USA	Infrared photoacoustic	CO_2_	0.005%
S-3A	AEI Technologies, Inc., Naperville, IL, USA	Solid ceramic oxide sensing element	O_2_	0.001%

**Table 2:** Injected, recovered gases and calculated recovery rates.

	*Hood 1*		*Hood 2*	
**Item**	**Ethanol (g)**	**Carbon Dioxide (ppm)**	**Ethanol (g)**	**Carbon Dioxide (ppm)**
Injected component	0.393	590	0.393	595
Total Flow rate (L/min)	615	615	607	607
Recovered component	0.383	576	0.387	591
**Recovery (%)**	**97.6**	**97.6**	**98.5**	**99.3**

**Table 3:** A comparison of unpublished methane emission estimates (g cow^−1^ day^−1^) with selected recent publications for lactating Holstein dairy cattle

**Publication**	**Ranges of CH_4_Emission rates (g cow^−^^1^ d^−^^1^)**	**Feed type**	**Ranges of Dry Matter Intake (kg cow^−^^1^ d^−^^1^)**	**Measurement Technique**
Unpublished data from current system	389–689	TMR (53.8% forage)	25.8–30.0	Ventilated-hood
[10]	224–274	TMR (36.2% forage)	27.5–28.5	Whole-animal environmental chamber
[11]	429–459	TMR (60% forage)	19.1–19.7	Ventilated-hood
[12]	273–352	Ryegrass pasture	15.4–17.8	SF_6_ method
[13]	241–293	TMR (45% forage)	18.0–19.4	Whole-animal environmental chamber
[14]	371–453	TMR (66% forage)	16.5–17.9	Whole-animal environmental chamber
